# The spectrum of imaging findings in takotsubo cardiomyopathy: a pictorial essay

**DOI:** 10.1590/0100-3984.2025.0019-en

**Published:** 2025-08-13

**Authors:** Camila Vilas Boas Machado, Roberto Sasdelli Neto, Gilberto Szarf, Walther Yoshiharu Ishikawa, Eduardo Kaiser Ururahy Nunes Fonseca

**Affiliations:** a,b,c,d,e Department of Imaging, Hospital Israelita Albert Einstein, São Paulo, SP, Brazil.

**Keywords:** Chest pain, Takotsubo cardiomyopathy, Cardiac magnetic resonance, Dor torácica, Cardiomiopatia de takotsubo, Ressonância magnética cardíaca

## Abstract

Takotsubo cardiomyopathy is an important differential diagnosis for acute chest pain. Imaging tests, such as ventriculography, echocardiography, computed tomography of the heart, and cardiac magnetic resonance, are valuable tools for diagnostic confirmation in this context. This study reviews the literature and exemplifies the spectrum of typical and atypical cardiac magnetic resonance findings in this disease, on the basis of the experience of our facility. Recognition of these characteristics underscores the roles that radiologists and cardiologists play in the care of patients with acute chest pain, enabling an accurate diagnosis and appropriate treatment.

## Abstract (pt)

A cardiomiopatia de takotsubo é um diagnóstico diferencial importante de dor torácica aguda. Exames de imagem, seja por ventri-culografia, ecocardiograma, tomografia computadorizada do coração ou ressonância magnética cardíaca, são ferramentas valiosas para a confirmação diagnostica nesse contexto. O presente estudo revisa a literatura e exemplifica o espectro dos achados típicos e atípicos dessa doença observados na ressonância magnética cardíaca, com base na experiência do nosso serviço. O reconhecimento dessas características reforça o papel do médico radiologista/cardiologista na linha de cuidado de pacientes com dor torácica aguda, possibilitando o diagnóstico e tratamento adequado.

## INTRODUCTION

Takotsubo cardiomyopathy, also known as broken-heart syndrome, stress-induced cardiomyopathy, and apical ballooning syndrome, was first described by Sano et al., in 1990^(1)^. Although its exact incidence is unknown, it has been observed in 1—2% of patients with suspected acute coronary syndrome, mainly affecting postmenopausal women^(1)^.

The clinical presentation of takotsubo cardiomyopathy resembles that of acute coronary syndrome, including precordial pain, dyspnea, electrocardiographic changes, and elevated troponin. One peculiar characteristic is that up to two thirds of patients can identify a physically or emotionally stressful event (Table 1) that occurred prior to the onset of symptoms^(2, 3)^.

**Table 1:** Examples of physical and emotional stressors that can trigger tako-tsubo cardiomyopathy.

Physical triggers	Emotional triggers
Stroke	Depression
Seizure	Divorce
Migraine	Post-traumatic stress disorder
Exacerbation of chronic pulmonary	Assault, robbery
obstructive disease	Change in employment
Pulmonary thromboembolism	Debt, bankruptcy
Gastrointestinal bleeding	Death of a family member
Incarcerated hernia	Arguments with family or coworkers
Pheochromocytoma	Environmental event (flood, earthquake, etc.)
Urinary lithiasis	
Labor/giving birth	Car accident
Chemotherapy	
Surgery/anesthesia	
Sepsis	

Although the pathophysiology of takotsubo cardiomyopathy is not completely understood, it is believed to be related to excessive catecholamine discharge, secondary to the stressful event, associated with differences in the density and distribution of adrenergic receptors in the myocardium. Reduced estrogen levels also appear to be involved^(1, 3)^.

Over the years, various diagnostic criteria have been proposed to help identify takotsubo cardiomyopathy in a patient complaining of acute chest pain. Here, we high-light those proposed by the Mayo Clinic^(4)^, which include the following:

- Transient hypokinesia, akinesia, or dyskinesia of the middle segments of the left ventricle (LV), with or without apical involvement, with segmental changes in contractility extending beyond a coronary territory, with or without an identified stressor stimulus
- Absence of obstructive coronary artery disease or angiographic evidence of acute plaque rupture
- New electrocardiographic changes (ST-segment elevation, T-wave inversion, or both) or slight elevation of troponin
- No pheochromocytoma or myocarditis.

From the time of diagnosis of takotsubo cardiomyopathy, cardiac magnetic resonance (CMR) has a well-established role in the evaluation of patients with the disease, allowing a detailed anatomical analysis, identification of segmental changes in myocardial contractility, and quantification of ventricular function, as well as the detection of edema and myocardial necrosis/fibrosis. It also allows the detection of complications, such as pericardial effusion, pleural effusion, dynamic obstruction of the LV outflow tract, acute pulmonary edema, intracavitary thrombus, and systemic thromboembolism. Finally, it is useful to exclude other pathologies with clinical presentations similar to that of acute coronary syndrome, such as myocarditis^(1)^.

Evaluation with CMR is also important for patient follow-up; it is recommended that it be performed three to six months after the acute event. In most cases, the prognosis is good, with normalization of segmental changes in contractility, recovery of the ventricular ejection fraction, and resolution of edema in the follow-up examination^(1)^.

## TYPICAL FINDINGS

**Figure 1:**
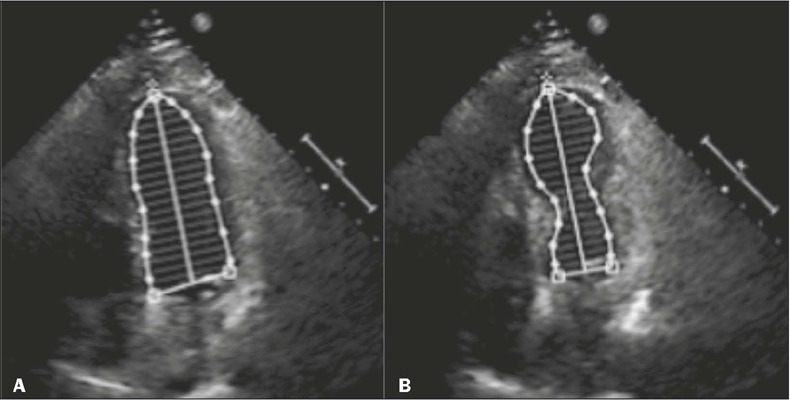
Four-chamber echocardiogram images, in diastole **(A)** and systole **(B),** showing hypokinesia in the apical region of the LV, together with hyperkinesia in the basal segments, resulting in the characteristic takotsubo morphology.

**Figure 2:**
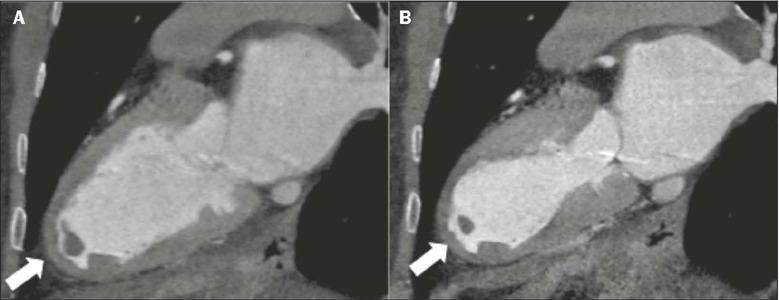
Coronary computed tomography angiography of a 78-year-old patient, reconstructed in a two-chamber view, in diastole **(A)** and systole **(B),** showing contractile alterations typical of takotsubo cardiomyopathy, leading to apical ballooning. Note the intracavitary thrombus in the apical region of the LV (arrows), a complication described in this pathology.

**Figure 3:**
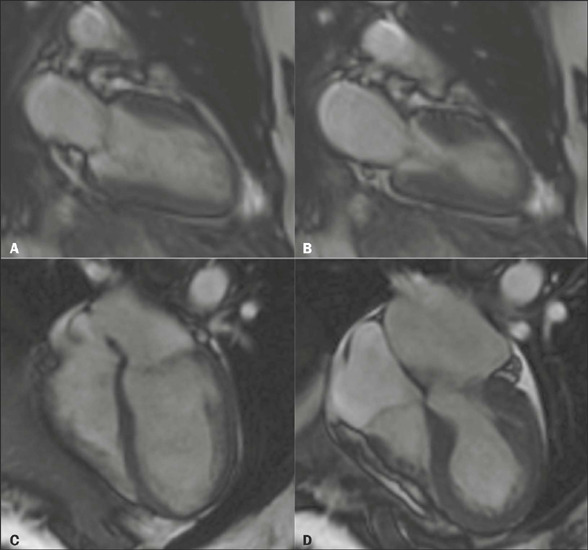
CMR images in the cine steady-state free precession (cineSSFP) sequence in two-chamber views **(A,B)** and four-chamber views **(C,D),** in diastole **(A,C)** and systole **(B,D),** showing the typical morphology of takotsubo cardiomyopathy.

**Figure 4:**
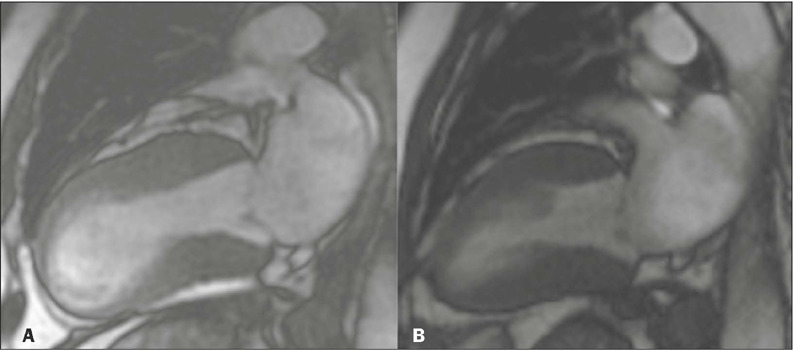
CMR images of a 62-year-old female patient, in the cine-SSFP sequence in two-chamber views in systole, demonstrating the apical ballooning of the LV typical of takotsubo cardiomyopathy **(A)** and the complete recovery of ventricular contractility in the follow-up examination performed five months later **(B).**

Classically, takotsubo cardiomyopathy is characterized by segmental changes in contractility—dyskinesia, hypokinesia, or akinesia of the mid-apical segments of the LV, accompanied by hyperkinesia of the basal segments—this combination resulting in the typical morphology^(1, 5, 6)^, as illustrated in Figures 1, 2, 3, 4 and described as similar to a jar used in Japan to capture octopuses, known as a takotsubo.

**Figure 5:**
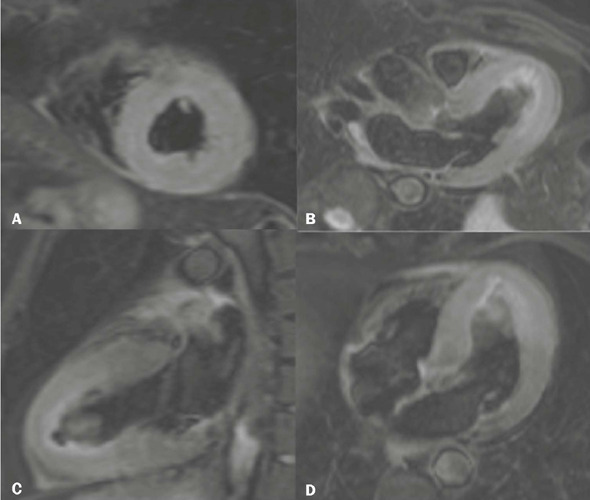
T2-weighted CMR (triple inversion recovery) sequence with fat saturation in a short-axis view **(A),** LV outflow tract view **(B),** two-chamber view **(C),** and four-chamber view **(D),** demonstrating marked edema in the mid-apical regions of the LV.

**Figure 6:**
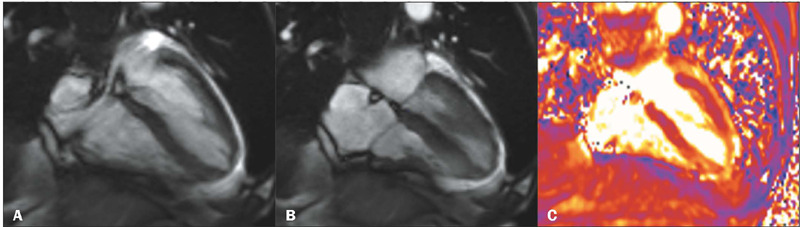
CMR images of a 91-year-old female patient, showing apical ballooning in a four-chamber cine-SSFP sequence, in diastole, **(A)** and systole **(B).** T2 map **(C)** demonstrating edema in mid-apical segments of the LV.

**Figure 7:**
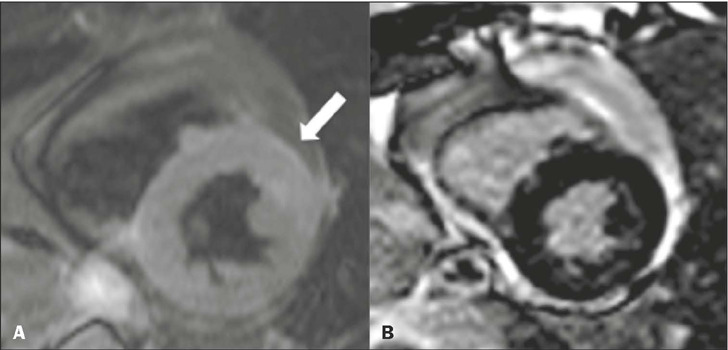
T2-weighted CMR sequences in short-axis views, showing edema in the anterior mid-LV segment **(A),** without correspondence in the delayed enhancement phase **(B).**

In addition to contractile changes, CMR allows the detection of myocardial edema, present in at least 80% of patients with takotsubo cardiomyopathy, traditionally on T2-weighted fast spin-echo sequences with triple inversion recovery (Figure 5) and, more recently, on T1 and T2 maps (Figure 6). In most cases, this edema is not accompanied by changes in the image on delayed enhancement sequences (Figure 7), unlike what happens in other conditions, such as acute myocardial infarction and myocarditis^(1, 5, 6)^.

## ATYPICAL FINDINGS

**Figure 8:**
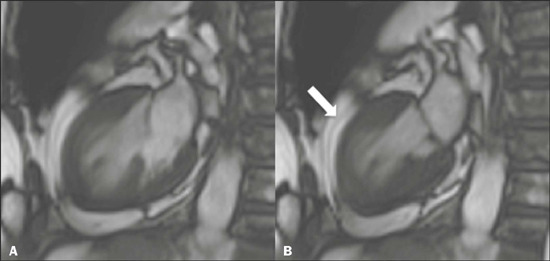
Cine-SSFP CMR images in two-chamber views, in diastole **(A)** and systole **(B),** demonstrating a takotsubo variant, characterized by marked hypokinesia of mid-ventricular segments (arrow in **B** ).

**Figure 9:**
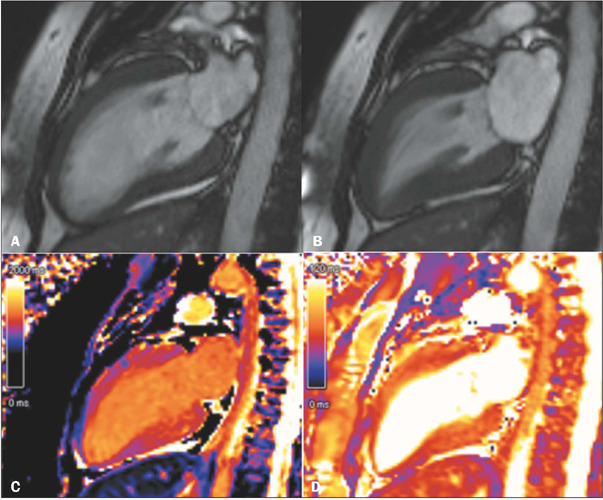
CMR images of a 41-year-old woman, showing marked hypokinesia of the anterior midventricular segments, together with relative hyperkinesia of the apical and basal segments **(A,B**—cine-SSFP sequence in two-chamber views in diastole and systole, respectively). The multiparametric T1 map **(C)** and T2 map **(D)** show increased intrinsic values in the hypokinetic segments, suggesting the presence of edema.

**Figure 10:**
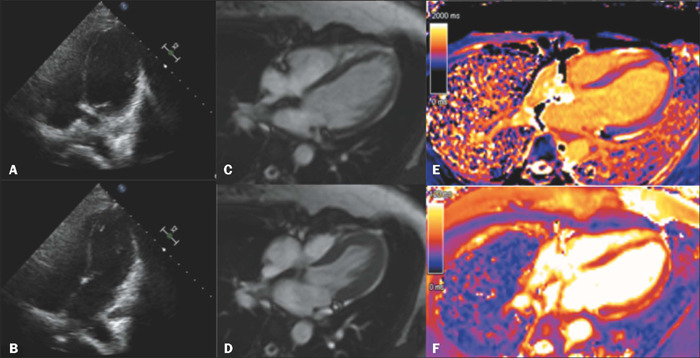
Echocardiogram and CMR of a 43-year-old female patient with chest pain after a seizure. The echocardiogram images **(A,B)** and the cine-SSFP CMR images **(C,D),** both in four-chamber views, demonstrate mid-ventricular segmental hypokinesia, particularly in septal segments. The T1 map **(E)** and T2 map **(F)** show diffuse edema, more pronounced in the hypokinetic segments.

**Figure 11:**
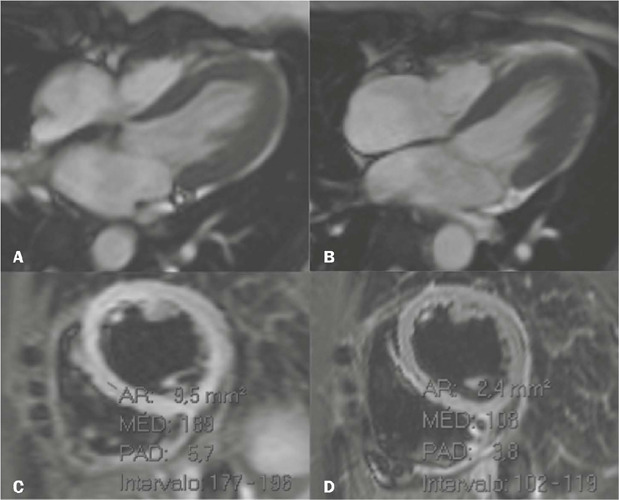
Images of the patient depicted in Figure 10. Cine-SSFP CMR sequences, in four-chamber views **(A,B),** and T2-weighted sequences with fat saturation **(C,D).** Images **A** and **C** show, respectively, the contractility changes and myocardial edema at the time of diagnosis. Images **B** and **D,** acquired one and a half months later, demonstrate the reversal of the findings.

The terms “atypical takotsubo cardiomyopathy” and “variant of takotsubo cardiomyopathy” are used when dyskinesia, hypokinesia, or akinesia affects nontraditional segments, sparing the cardiac apex and involving basal or mid-ventricular segments (Figures 8, 9, 10, 11), which occurs in up to 40% of cases^(4, 5)^.

**Figure 12:**
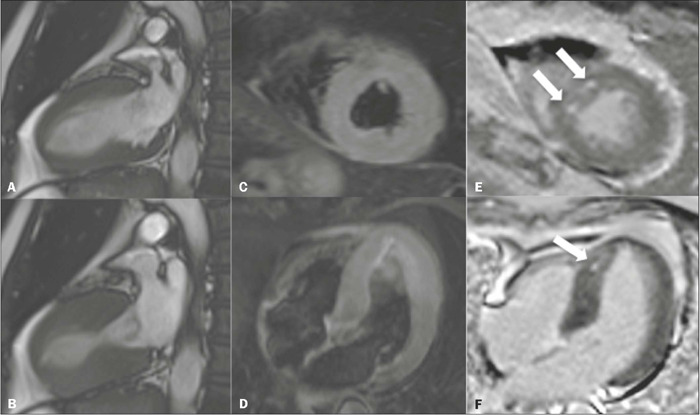
CMR examination of a 66-year-old male patient with typical segmental changes in contractility in cine-SSFP sequences in two-chamber views **(A,B),** midapical myocardial edema in the LV on the T2-weighted images in a short-axis view **(C)** and four-chamber view **(D),** together with small foci of delayed enhancement with a non-ischemic pattern, indicated by the arrows in the short-axis view **(E)** and four-chamber view **(F).**

In addition to unusual segmental changes in contractility, a small proportion of patients may also present with delayed myocardial enhancement (Figure 12), which manifests as small, scattered foci with a clearly non-ischemic pattern, often identified only by quantitative analysis with the aid of software and not persisting in follow-up examinations performed a few weeks after the acute event.

Although the pathophysiology of that finding is not yet fully understood, it is believed to be related to changes in proteins (collagen type 1) in the myocardium during the acute phase of the disease, and not to myocardial necrosis or fibrosis^(1)^.

The clinical significance of the variants of takotsubo cardiomyopathy is still an open question. Some clinical differences, such as the involvement of women of a slightly younger age (mean of 62 years) and the association with neurological diseases, have been described. Depression of the ST-segment, lower B-type natriuretic peptide values on admission, and less pronounced changes in the left ventricular ejection fraction are also characteristics that stand out in these cases^(7, 8, 9)^.

## CONCLUSION

Takotsubo cardiomyopathy is a diagnosis that should be considered in the context of chest pain in an emergency care setting. The use of CMR allows noninvasive diagnosis, providing information additional to that obtained by echocardiography and enabling the detection of any complications^(10, 11)^.
